# Silicon Nanodisk Huygens Metasurfaces for Portable and Low-Cost Refractive Index and Biomarker Sensing

**DOI:** 10.1021/acsanm.1c04443

**Published:** 2022-03-16

**Authors:** Isaac O. Oguntoye, Brittany K. Simone, Siddharth Padmanabha, George Z. Hartfield, Pouya Amrollahi, Tony Y. Hu, Adam J. Ollanik, Matthew D. Escarra

**Affiliations:** † Department of Physics and Engineering Physics, Tulane University, New Orleans, Louisiana 70118, United States; ‡ Center of Cellular and Molecular Diagnostics, Tulane University, New Orleans, Louisiana 70112, United States; § Department of Physics, University of Colorado Boulder, Boulder, Colorado 80309, United States

**Keywords:** dielectric metasurfaces, nanoantenna arrays, nanophotonics, microfluidics, portable biosensor, refractive index sensing, biomarker detection

## Abstract

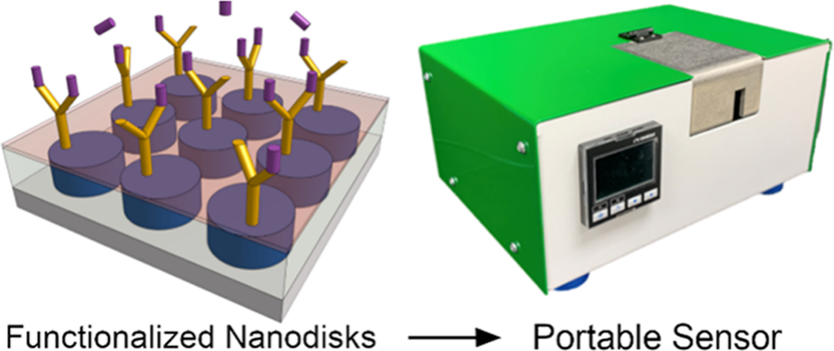

Biomarker detection and bulk refractive index sensing are important across multiple industries ranging from early medical diagnosis to chemical process quality control. The bulky size, high cost, and complex architecture of existing refractive index and biomarker sensing technologies limit their use to highly skilled environments like hospitals, large food processing plants, and research labs. Here, we demonstrate a compact and inexpensive refractive index sensor based on resonant dielectric photonic nanoantenna arrays or metasurfaces. These dielectric resonances support Mie dipole and asymmetric resonances that shift with changes in their external environment. A single-wavelength transmission measurement in a portable (<250 in.^3^), low-cost (<$4000) sensor shows sensitivity to 1.9 × 10^–6^ change in the fluid refractive index without the use of a spectrometer or other complex optics. Our sensor assembly allows for measurements using multiple metasurfaces with identical resonances or varying resonance types for enhanced diagnostics on the same chip. Furthermore, a 10 kDa culture filtrate peptide CFP-10, a marker for human tuberculosis, is detected with our sensor with 10 pM resolution. This system has the potential to enable facile, fast, and highly sensitive measurements with adequate limits of detection for personalized biomedical diagnoses.

## 1 Introduction

Refractive index sensing has garnered much attention due to its usefulness in determining fluid concentration, food contamination, biomedical diagnosis, and trace gas detection.^1^ The ability of light to change its speed as it traverses from one medium to another represents an important measurement useful for characterizing the composition of a bulk fluid.^2^ Conventional refractive index sensing platforms are large and expensive, posing a limitation to scalable deployment and applicability. Many of these are based on complex instrumentation or beam propagating mechanisms such as prisms, interferometers, spectrometers, and optical fibers.^3^ Their large and complex form makes them difficult to implement for portable and robust on-chip device integration, thus limiting the usability of these systems to highly skilled technicians in the industry or research labs. These include surface plasmon resonance (SPR)-based sensors, relying on electromagnetic field oscillations along the interface between a metal and a dielectric e.g., prism-coupled Kretschmann-structured sensors,^4−6^ localized surface plasmon resonance (LSPR)-based sensors,^7−9^ and fiber gratings.^10^ Also, LSPR- and SPR-based sensing methods have been combined in plasmonic nanohole arrays, utilizing their extraordinary transmission for refractive index sensing.^11,12^ Each of these methods presents a unique way of tracking changes in the refractive index of fluids. Surface plasmon resonance-based sensing has been extended for use in biosensor applications in the industry through implementation in the Biacore system. Although this method has proven to be highly sensitive, it also relies on complex optics for functionality.^13,14^ The highly absorbing nature of plasmonic materials at visible and infrared wavelengths could limit the application of these sensors due to absorption-induced heating.^15^ Interferometric-based sensors have been developed and studied extensively for plasmonic platforms;^16^ many of these methods, while highly sensitive, still require the use of external spectroscopy for characterization.^17^ Additionally, two-dimensional materials have been utilized for photonic-enhanced refractive index sensing, namely, graphene and similar materials with high chemical stability and biocompatibility.^18^ These materials are typically integrated into plasmonic sensing platforms, implying the same drawbacks of cost and complexity. Dielectric nanoparticles and nanoresonator arrays are preferred over their plasmonic counterparts as the antenna material for our metasurfaces due to their low absorption losses, and thus high efficiency, in the near-infrared regime of the electromagnetic spectrum.^19^ Many platforms have been demonstrated using dielectric-based structures designed to locally interrogate refractive index changes in their environment. Examples include Bloch surface wave-based photonic crystal sensors. While achieving a high resolution, the propagating Bloch surface wave (BSW) platform depends on a prism and spectrometer for spectrally resolving local refractive index variations. Recently, a simple common path interferometric-based sensor was demonstrated; however, the optics components used present challenges for compact device integration.^20^ The aim of this work is to present an all-in-one device utilizing a low-cost dielectric metasurface-based resonant platform for highly sensitive bulk fluid refractive index sensing. This same approach can be used for biomolecule detection, where the adsorption of the biomarker of interest onto the metasurface similarly disturbs the resonances and corresponding transmission.

To validate its performance, this sensor is demonstrated to accurately measure tuberculosis (TB), one of the top 10 most deadly diseases worldwide. While the number of people with access to preventive treatment has grown in recent years, access to quality and prompt detection is still a challenge. The World Health Organization predicted that a 50% drop in TB detection over a period of 3 months could have resulted in 400,000 additional deaths in countries with high TB cases in 2020.^21^ This, as well as the recent global COVID-19 pandemic, has accentuated the need for accurate, fast, inexpensive, and reliable methods for label-free biomolecule detection in the world today. Although some demonstrations of label-free resonant photonic biosensors have been done for various biomolecules such as streptavidin,^15^ immunoglobin G antibody,^22^ and prostate-specific antigen,^13^ most efforts have been made toward proof-of-concept demonstrations and not so much on the implementation of a novel photonic sensing method in a low-price and portable sensor. Here, we design and assemble a compact and low-cost optoelectronic sensor for bulk and surface refractive index sensing as well as TB biomarker surface analyte detection using a dielectric nanodisk array platform.

## 2 Design Methodology

### 2.1 Principle of Refractive Index Sensing

**Figure 1:**
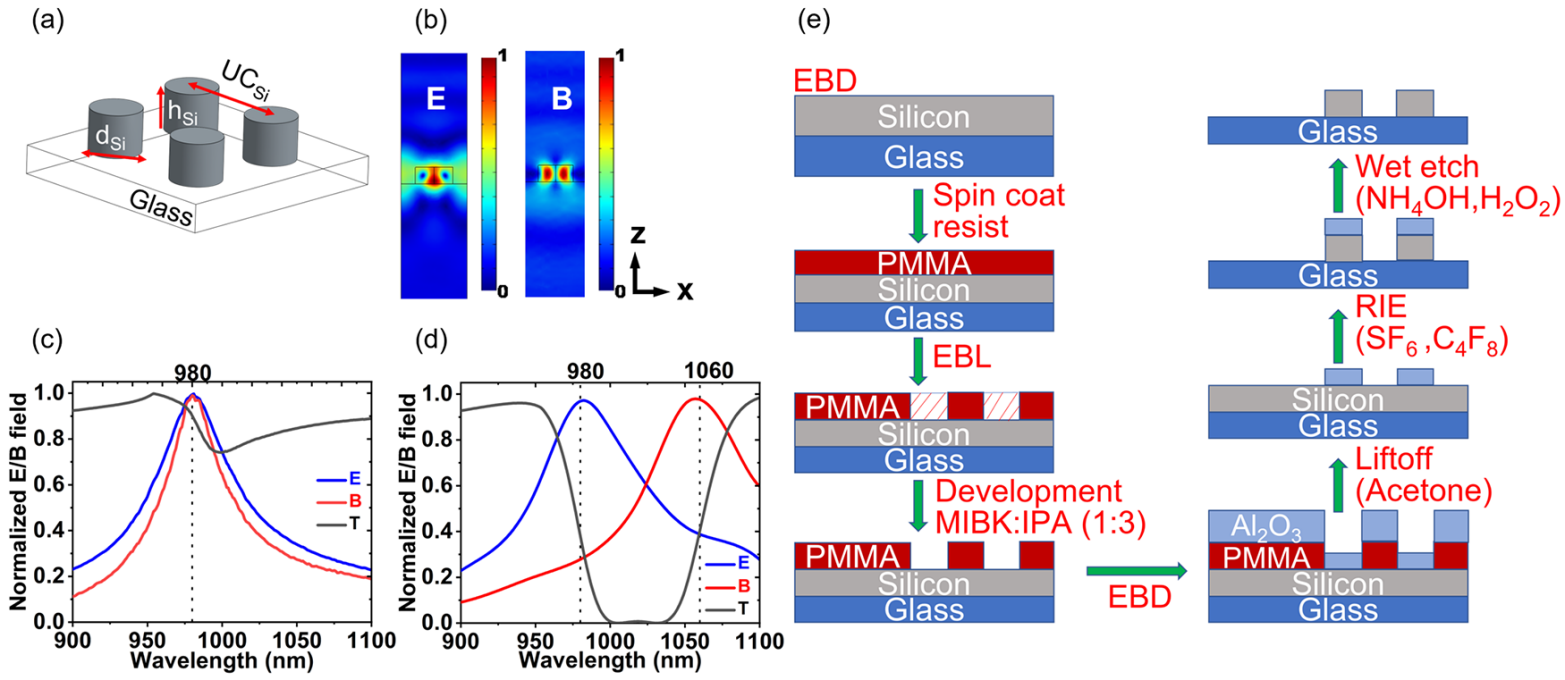
(a) Schematic of a portion of the dielectric nanoantenna array composed of amorphous silicon resonators on a glass substrate. The dimensions shown are diameter (*d*_Si_, 330 nm), height (*h*_Si_, 190 nm), and unit cell periodicity (UC_Si_, 581 nm). (b) Field profiles for the electric field (E) and magnetic field (B) confinement in nanodisk meta-atoms at resonance. The field outside the nanodisk signifies the nearest-neighbor interaction in a periodic array. (c) Spectrally overlapping electric and magnetic dipole resonances are shown for a highly transmissive dielectric metasurface. (d) Spectrally misaligned electric and magnetic dipole resonances are shown for a metasurface with a strong reflectance peak. (e) Schematic showing fabrication protocol for silicon nanodisk arrays (electron beam deposition, EBD; electron beam lithography, EBL; reactive ion etching, RIE; poly(methyl methacrylate), PMMA; aluminum oxide, Al_2_O_3_; sulfur hexafluoride, SF_6_; octafluorocyclobutane, C_4_F_8_; ammonium hydroxide, NH_4_OH; hydrogen peroxide, H_2_O_2_).

The strong forward or backward scattering in dielectric subwavelength nanoantennas is possible due to the simultaneous excitation of electric and magnetic dipole modes. Obtaining high scattering efficiencies in either direction is achieved by the interference of these resonant modes. Here, we illustrate our dielectric nanoantenna array based on amorphous silicon on a glass substrate. The structure and dimensions of this dielectric platform are shown in Figure 1a. These dielectric nanoantenna arrays, or Huygens metasurfaces, can be engineered such that the electric and magnetic dipole resonances are spectrally aligned, as shown in Figure 1c, leading to near-unity transmittance at the resonant wavelength. On the contrary, a careful spectral misalignment of the electric and magnetic dipole resonances leads to high reflectance, resulting from these destructively interfering modes^23^ (Figure 1d). The slope of the metasurface-induced reflectance peak, in addition to the spectral shift of this peak caused by varying the encapsulant index, makes it possible to perform single-wavelength measurements using transmittance shift as the detected optical response. This may be accomplished without any deviation in the optical path and without the use of complex or expensive optics, such as a spectrometer. The low-loss nature of these dielectric metasurfaces makes them preferable to their plasmonic counterparts. This resonant interaction is accompanied by coupling to their nearest-neighbor antennas. This coupling leads to undesirable effects in certain static applications such as beam deflectors, where phase-gradient metasurfaces are used. However, it is a strong advantage for applications such as surface analyte detection and bulk fluid sensing involving photon–matter coupling in the metasurface surroundings.^24,25^ The evanescent field around the resonators is sensitive to changes in the refractive index of the nanoantenna environment (Figure 1b). As a further advantage, these Huygens metasurfaces have a low aspect ratio, allowing easy fabrication and precise engineering of their dimensions to achieve electric and magnetic dipole resonances designed to interact with specific fluids and biomolecules (fabrication steps are shown in Figure 1e). They can also excite asymmetric resonances leading to enhanced sensitivity. The electric dipole resonance is more sensitive to encapsulant index variations for the Mie resonances, while both electric and magnetic resonances are sensitive to the encapsulant index variations for the asymmetric resonances (see Figure S-1). The light–matter interactions in these metasurfaces depend on the size and shape of the meta-atoms as well as their material properties.^26^

### 2.2 Metasurface Design

**Figure 2:**
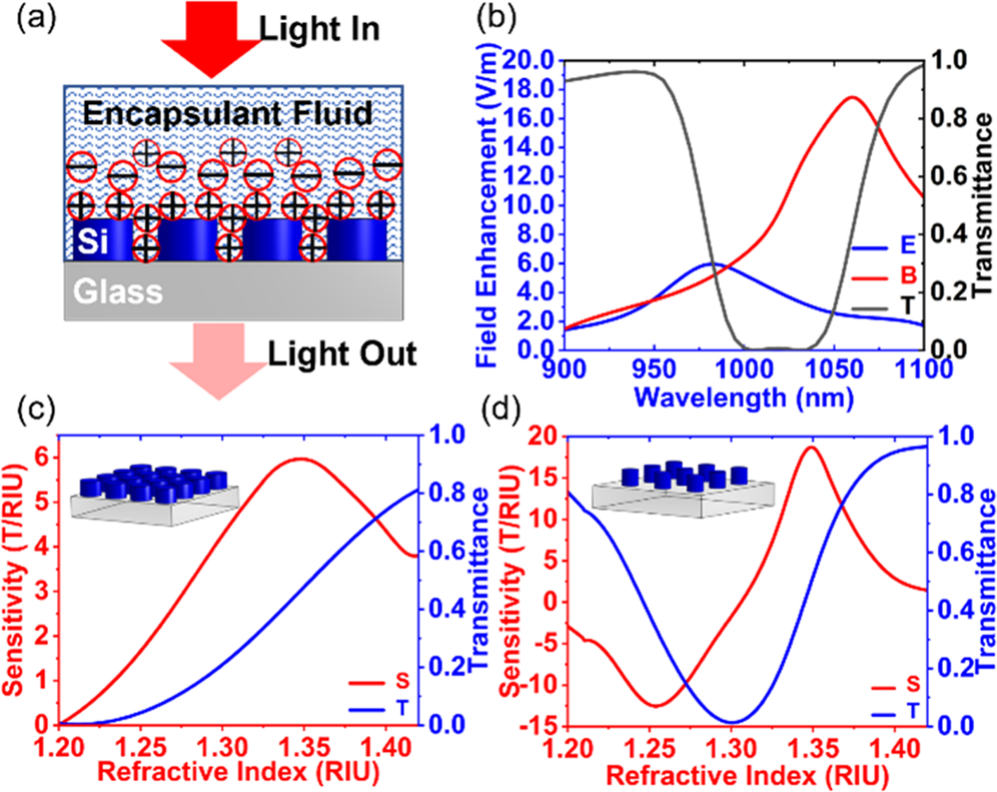
(a) Schematic showing the encapsulant fluid, nanoantenna, and substrate domains. Light is incident from the top, interacts with the molecules of the fluid creating an electric double layer, and the optically induced response is transmitted through the glass and collected on a detector. (b) The resulting reflectance peak (black) from spectrally shifting electric (red) and magnetic (blue) fields. Field enhancement is calculated as the amount by which the electric and magnetic fields are enhanced due to the presence of the silicon metasurface relative to no metasurface. (c) Metasurface design one: Mie resonance nanodisks with a maximum sensitivity of 5.8 T/RIU. (d) Metasurface design two: asymmetric resonance nanocylinders with a maximum sensitivity of 20 T/RIU.

The metasurface-based chip measurement setup is illuminated with light impinging at normal incidence on the chip in the presence of an encapsulating fluid (Figure 2a), which is varied to demonstrate the sensing capabilities of the chip. This metasurface is designed using finite element modeling (COMSOL Multiphysics) to predict the optical response of the chip before fabrication. A strong reflectance peak is obtained when the electric and magnetic dipole resonances are spectrally separated by 80 nm (Figure 2b). Device sensitivity can be defined in multiple ways as established in a previous work.^27^ Here, we use the definition of sensitivity as the T/RIU—change in transmittance (*T*) per change in one refractive index unit (RIU).

A single-wavelength laser passes through three metasurfaces and one reference (no-metasurface) channel, and the corresponding transmission is measured by a four-quadrant photodetector. This merging approach may use three of the same metasurface for improved data averaging and fidelity. Or, taking advantage of the responsiveness of our Huygens metasurface platform to geometric changes, the sensor may utilize three different metasurface designs. Multiple simultaneous measurements on the same chip with different metasurface designs demonstrate the versatility of our sensing method as, for example, it enables us to measure fluid samples over a wider refractive index range than is possible with a single metasurface. In this work, we design and make three Mie resonance metasurfaces on a single chip. As reported in a previous work, this type of metasurface demonstrates an experimental sensitivity and figure of merit (FOM) of 323 nmRIU^–1^ and 5.4, respectively.^28^ Each of these three metasurfaces is designed to have the same resonant behavior, yielding a peak sensitivity of 5.8 T/RIU (Figure 2c) and an FOM of 2.1 (Figure 2b). We also design and make two different metasurfaces in three channel slots—one channel for the Mie resonance metasurface and the other two channels for the asymmetric resonance nanocylinder arrays. This results in a strong and more sensitive device, thus obtaining a sensitivity of 20 T/RIU for the asymmetric resonance metasurface (Figure 2d) and an FOM of 11.1 (see Figure S-1).

### 2.3 Sensor Design

**Figure 3:**
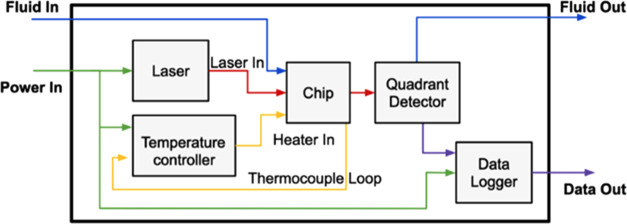
Nanophotonic microfluidic sensor block diagram with associated inputs and outputs. The blue path is the sample fluid stream, the green path is the electrical power, the red path is the laser light stream, the yellow path is the heater loop, and the purple path is the data output.

Taking advantage of resonant metasurfaces with a distinct spectral reflectance peak allows for highly sensitive single-wavelength measurements using inexpensive optics. The use of a small data logger and a proportional-integral-derivative (PID) temperature controller to reduce the size and cost of computational equipment enables a highly competitive refractive index sensor at a fraction of the cost. The produced system is a result of integrating the metasurfaces into a microfluidic chip that is interrogated by a simplified optical system. The full system diagram showing all inputs, outputs, and subfunctions is given in Figure 3. The sample fluid is injected through exterior tubing before passing through the microfluidic measurement chip and then out of the sensor. These three systems (microfluidic, optical, and electrical) come together within a single integrated housing and produce output voltage data that corresponds to relative transmittance, an indicator for refractive index change.

### 2.4 Microfluidic Design

**Figure 4:**
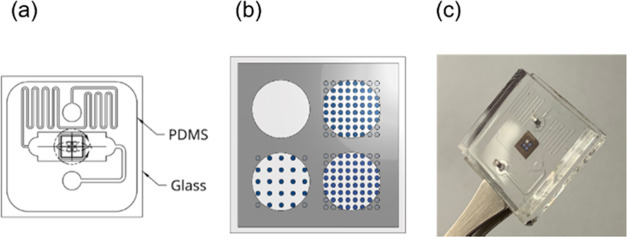
(a) Microfluidic diagram showing the fluid inlet (top circle), temperature stabilization channel, the main testing channel with the optically active area, and a fluid outlet (bottom circle). Fabrication processes are included in the Supporting Information. (b) Diagram showing three different metasurfaces and a reference channel with the overlaid reflectance mask. (c) Sensing chip showing three 500 micron square metasurfaces and a blank reference within a chromium reflectance mask. These are fabricated on a microscopic glass slide and bound within a PDMS microfluidic channel. The fluid inlet and outlet are shown above and below the chromium mask, respectively.

Microfluidic channels facilitate changing the metasurfaces’ encapsulating layer and therefore the resonant response of the metasurfaces.^29^ The inlet path length of the microfluidic channel is maximized before entering the measurement stage to allow the fluid temperature to equilibrate with that of the surrounding glass and PDMS (Figure 4a). Sample fluid then passes over a microfabricated reflectance mask (Figure 4b), designed to split a single incident beam into four: three metasurface sample beams and one reference beam where no metasurface is present. The reflectance mask is also intended to prevent the impinging beam from reaching the photodetector without first passing through the metasurfaces. This design allows up to three separately tuned metasurfaces and a reference measurement to be taken simultaneously, accounting for noise in the measured signal due to fluctuations in ambient temperature, liquid pressure, and the intensity or wavelength of the laser. Figure 4c shows a fabricated metasurface measurement chip with microfluidic channels.

### 2.5 Electronics and Optics Design

**Figure 5:**
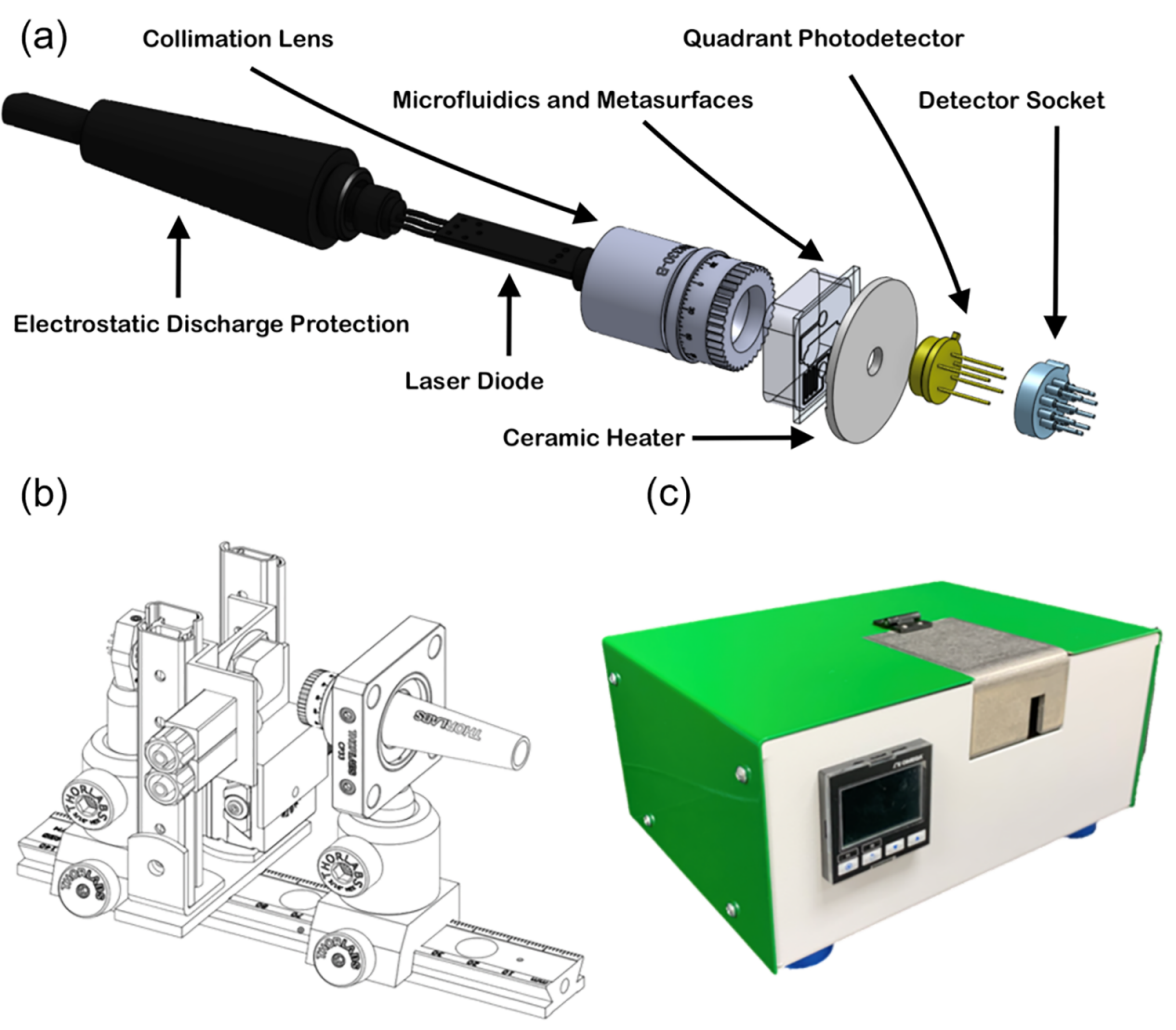
(a) Exploded view of the optical train aligned to the metasurface-based measurement chip. (b) Schematic of the optical system and microfluidic chip stage with mounting and alignment components. (c) Image of the fully functional, portable sensor.

All metasurface dimensions are designed for maximum T/RIU at a wavelength of 980 nm, so that the sensor can employ affordable, off-the-shelf parts. A Thorlabs L980P010 Laser Diode is chosen as the light source, which is collimated and incident upon the microfluidic measurement chip. This diode was characterized using the Ocean Optics NIRQuest spectrometer to verify an operating wavelength of 980 nm. The outgoing light beams are then collected by a first sensor QP5.8-6-TO5 quadrant photodiode, containing four distinct active areas corresponding to each beam. The optical train is shown schematically in Figure 5a. All optics are aligned and positioned with standard Thorlabs dovetail rails, mounts, and posts (see Figure 5b). The voltages for each active area are logged in a Delphin Loggito USB Data Logger using the included Profilog software package for analysis.

All electronic components within the sensor are powered through a 24 V (DC) wall adapter that connects to the housing on the backside of the enclosure. Twenty-four volts is passed to an Omega CND3 PID controller for temperature regulation, and in parallel, the voltage is stepped down through a series of cascading voltage regulators to meet the power requirements of a Thorlabs LD1100 constant power laser diode driver (see the circuit diagram in Figure S-3). The PID controller regulates the measurement chip temperature to within ±1 °C based on the feedback from a K-type thermocouple. This is accomplished using a ceramic heater modulated by a relay switch (see Figure S-3) placed underneath the glass substrate of the measurement chip; the heater has a 4.0 mm diameter hole in the center to pass the transmitted light to the quadrant photodetector.

Temperature control is critical to minimize noise-based uncertainty in measurement resolution, for example, maintaining the metasurface and reference channels at the same temperature during measurements.

### 2.6 Sensor Prototype Assembly

A custom housing (*L* = 8.5″, *W* = 6.5″, *H* = 4″) containing all required equipment developed for this sensor testbed was manufactured by Protocase to prevent ambient light from interfering with measurements and to promote portability (Figure 5c). Vibration-damping feet on the bottom of the enclosure reduce exterior mechanical noise. Cutouts are included for the through-wall PID temperature controller, power supply inlet, and USB connector for data output. In addition to a removable cover, a hinged door provides access to the measurement stage for optical alignment and quick measurement chip replacement. The microfluidic/metasurface measurement chip itself is precisely positioned via a small manual two-axis micrometer stage to ensure proper alignment. The inlet and outlet ports for the sample liquid are syringe-compatible and are affixed close to the laser path to minimize required sample volumes. An external container collects the output fluid on the completion of testing.

## 3 Experimental Methodology

### 3.1 Bulk Fluid Sensing

For solution refractive index and composition measurements, demonstrations are done using saline at varying concentrations. The sample fluid is introduced into the microfluidic chip by a syringe via the inlet tubing. The measurement is taken while the solution is at rest within the channel to ensure that there is no fluctuation introduced by flow-induced pressure changes. The sample fluid then passes through the outlet tubing, and a pocket of air is introduced to flush out any remaining fluid. The channel is cleaned with deionized water to remove any residue left in the channel. Deionized water is measured first as the zero-concentration baseline to which all changes in transmittance (Δ*T*) values are referenced. Photodetector voltage data is collected over a period of 2 min. The reference (no-metasurface channel) beam is used to normalize into transmittance using the equation
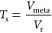
1here, *T*_s_ is the relative transmittance, *V*_meta_ is the photodetector voltage output from the metasurface incident beam, and *V*_r_ is the reference beam voltage. The resultant transmittance values are relative and not absolute, and thus, the quantity of interest in finding refractive index is not transmittance but a normalized change in transmittance from the zero-concentration solution.

At lower concentrations, the sodium cations (Na^+^) in solution are preferentially adsorbed to the surface terminating the hydroxyl anion (OH^–^) bond on the silicon surface. This explains the relatively significant change in the sensor’s optical response for very small changes in the refractive index due to the changing effective encapsulant refractive index surrounding the metasurfaces. This surface adsorption phenomenon results in a nonlinear relationship between the transmitted signal and saline concentration and enables sensor performance with a lower detection limit. This nonlinear behavior occurs for concentrations of saline below 10^–2^ M. The surface adsorption feature, illustrated in Figure 2a, is in agreement with the electrical double layer theory where co-ions are repelled and counterions are attracted to a charged surface.^30,31^

When higher saline concentrations flow through the sensor, the sites available for surface adsorption become quickly saturated. The influence of bulk encapsulant refractive index changes begins to dominate resulting in a linear trend. The following equation is then used to convert Δ*T* values into refractive index measurements for a linear system
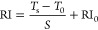
2Here, RI is the refractive index of the sample solution, *T*_0_ is the same relative transmittance calculation (eq 1) but for a zero saline concentration solution, *S* is the bulk metasurface sensitivity in units T/RIU, and RI_0_ is the refractive index value of the zero-concentration base solution. eq 2 is used to calculate the linear sensitivity (*S*) of the sensor. A known empirical relation for saline’s refractive index change with concentration and temperature is used to set the RI_0_ and RI values for the bounds of the measured range.^32,33^ With this information, by rearranging the terms in eq 2, S can be solved for and used to find RI for all intermediate values in the linear operating range.

### 3.2 Biomarker Detection

Detection of biomarkers of interest, such as those associated with infectious diseases, may be accomplished with the same sensor by utilizing a biochemical assay to perform functionalized surface sensing as opposed to the bulk fluid sensing discussed before. The assay procedure ensures selectivity through antibody–antigen interactions, where the antibodies are bound to the surface-functionalized metasurface as a capture site and antigens from the sample solution attach during the measurement phase. Sensor optical measurements are taken at each step of the assay to track the change in transmission caused by each component. The complete assay can be seen in Figure 7a–e, with further details given in the Supporting Information.^34−39^

## 4 Results and Discussion

### 4.1 Sensor Performance

**Figure 6:**
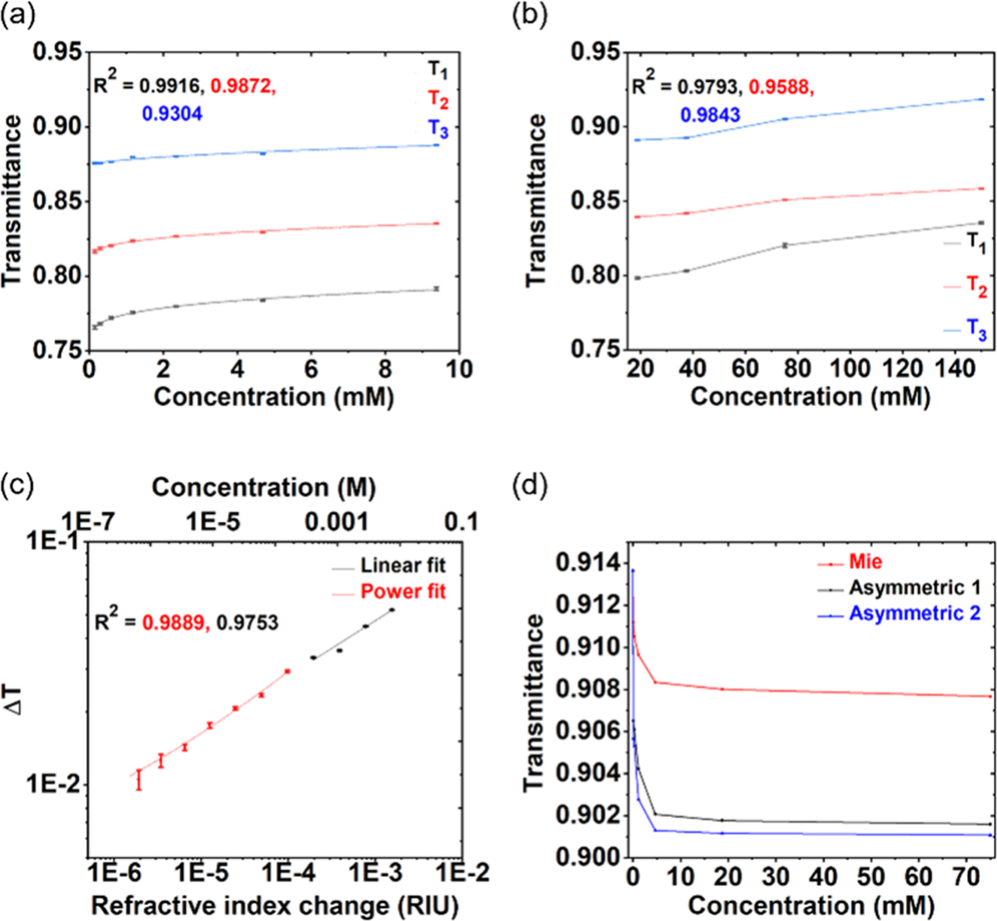
Transmittance vs concentration measurements for three similar metasurfaces with saline solutions showing a similar repeatable trend across the three metasurfaces for (a) low saline concentrations (<∼10^–2^ M) and (b) high saline concentrations (>∼10^–2^ M). (c) LOD plot for one Mie dipole resonance metasurface for a broad range of saline concentrations. Δ*T* represents the relative transmittance change. (d) Transmittance vs. saline concentration for one Mie dipole resonance and two asymmetric resonance metasurfaces measured on the same chip. Red is the Mie nanodisk array; the other two are nanocylinder arrays with different lateral dimensions supporting asymmetric resonances.

Figure 6a,b shows the measured optical response data collected for saline solutions with varying concentrations. Results indicate a 9849 *R*^2^ linear fit for an experimental standard curve consisting of 11 points (excluding water) representing saline concentrations varying from 18.9 mM to 150 mM. We define the sensor limit of detection (LOD) in two ways. First, the concentration LOD (LOD_conc_) describes the smallest concentration of solution below which the sensor can detect no change in optical response. Also, the refractive index LOD (LOD_RI_) represents the smallest change in the effective encapsulant refractive index that can be detected by our sensor because of solute concentration change from one fluid to another. From system noise analysis (see Table 2), we expect to see a theoretical LOD_RI_ of about 1 × 10^–6^ RIU, as limited by the data logger resolution and our current level of noise control. Here, we demonstrate a measured LOD_conc_ of 7.3 × 10^–5^ M (4272.8 ngmL^–1^) corresponding to an LOD_RI_ of 1.9 × 10^–6^ RIU using the aforementioned empirical relation. The obtained LOD is compared with state-of-the-art dielectric-based photonic sensors in Table 1. Figure 6c shows the data on a log plot, illustrating the LOD for this sensor’s bulk refractive index measurements.

**Table 1:** Dielectric-Based Photonic Sensor Types and Their Limits of Detection in Units of RIU^a^

sensor type	interferometer-based	photonic crystals	Bloch surface waves	photonic crystal cavity	whispering gallery mode	dielectric nanodisks (this work)
LOD [RIU]	1.8 × 10^–6^ (ref (20))	1.97 × 10^–6^ (ref ^40^)	3.8 × 10^–6^ (ref ^41^)	7.8 × 10^–6^ (ref ^42^)	4.5 × 10^–6^ (ref ^43^)	1.9 × 10^–6^

a The low-cost dielectric metasurface-based sensor demonstrated here has an LOD comparable to these state-of-the-art refractive index sensors.

The device design approach (three metasurface slots and one reference channel slot measured simultaneously) enables the measurement of three similar or three different metasurfaces at a time. Testing three similar metasurfaces serves to enhance measurement fidelity and assess reproducibility, whereas a chip with different metasurfaces expands the range of testable fluid refractive indices and increases system measurement flexibility. This can be seen in Figure 6d, where we show an increasing sensitivity for the low-concentration region for bulk saline solutions when measured with asymmetric resonance metasurfaces vs Mie resonance metasurfaces. Furthermore, optical transmittance decreases with increasing saline concentration, showing a different trend from our earlier measured metasurface results in Figure 6a. This can be attributed to a mismatch between fabricated geometries and modeled geometries, where the transmission band has red-shifted such that these measurements occur at a different spectral location relative to that transmission band, with an opposite change in transmittance vs. concentration.

### 4.2 Sensor Noise Analysis

System noise is a limiting factor in achieving a competitive LOD. Refractive index is dependent upon temperature^44^ and pressure.^45^ As shown in Table 2, we have demonstrated control over these two parameters to measure refractive index changes (Δ*n*) on the order of 10^–4^ RIU without continuously averaging to reduce environmental perturbation effects. Taking advantage of simultaneous referencing eliminates the impact of drift from temperature, pressure, and even incident power and wavelength fluctuations. Vibrational noise is more difficult to characterize and mechanically stabilize in a portable sensor, but utilizing a rolling average along with a reference channel that experiences the same mechanically induced fluctuation has proven effective enough to allow for refractive index changes to be detectable down to ∼10^–6^ RIU. The main factor that limits the achievement of lower LOD is the cost of higher precision data loggers, which we avoid maintaining competitive sensor total cost. A lower noise limit exists due to the resolution of the Delphin Loggito USB Logger used for data collection of voltage signals at 01% of the range. In our case, the maximum signal is ∼0.250 V, leading to a 2.5 × 10^–5^ V resolution before averaging or normalization.

**Table 2:** Noise Analysis Table Defining Metrics Needed to Achieve Acceptable Control at Three Different Sensitivity Levels: 10^–4^ Δ*n*, 10^–6^ Δ*n*, and 10^–8^ Δ*n*^a^

noise analysis	metric	current possibilities (Δ*n*)	control needed (Δ*n* ∼ 10^–4^)	control needed (Δ*n* ∼ 10^–6^)	control needed (Δ*n* ∼ 10^–8^)
detector/power	∼3.7 × 10^–14^ RIU·mW^–1^	∼10^–8^	10^10^ mW	2.7 × 10^7^ mW	10^6^ mW
vibrational	∼2.9 × 10^–3^ RIU·nm^–1^	∼10^–4^	3.5 × 10^–2^ nm	3.5 × 10^–4^ nm	3.5 × 10^–6^ nm
temperature	10^–4^ RIU·°C^–1^	∼10^–5^	1 °C	10^–2^ °C	10^–4^ °C
pressure	10^–5^ RIU·atm^–1^	∼10^–8^	10 atm	10^–1^ atm	10^–3^ atm
wavelength	10^–5^ RIU·nm^–1^	∼10^–6^	10 nm	10^–1^ nm	10^–3^ nm
data logger	5 digits for 10^–6^	∼10^–6^	3 digits	5 digits	7 digits

a With our current level of control of each of these factors, a sensitivity of Δ*n* ∼ 10^–4^ can be achieved with simultaneously referenced data. Referencing and data averaging are needed to attain Δ*n* ∼ 10^–6^. Achieving temperature control and data logger precision to obtain Δ*n* ∼ 10^–8^ is currently beyond the scope of our sensor.

A complete bill of materials, as given in the Supporting Information, places our current sensor cost for one unit at $3994. In comparison to equally sensitive technologies, our price point per unit is 87–96% lower.^28^ This is possible due to the simplicity of the required equipment for the sensor described here as compared to other established methods.^46^ Implementing more accurate controls and data acquisition would decrease the current LOD while increasing sensor cost. This could also be done through incorporation of a microcontroller and simple display to directly output data from the device.

### 4.3 Biomarker Detection Results

**Figure 7:**
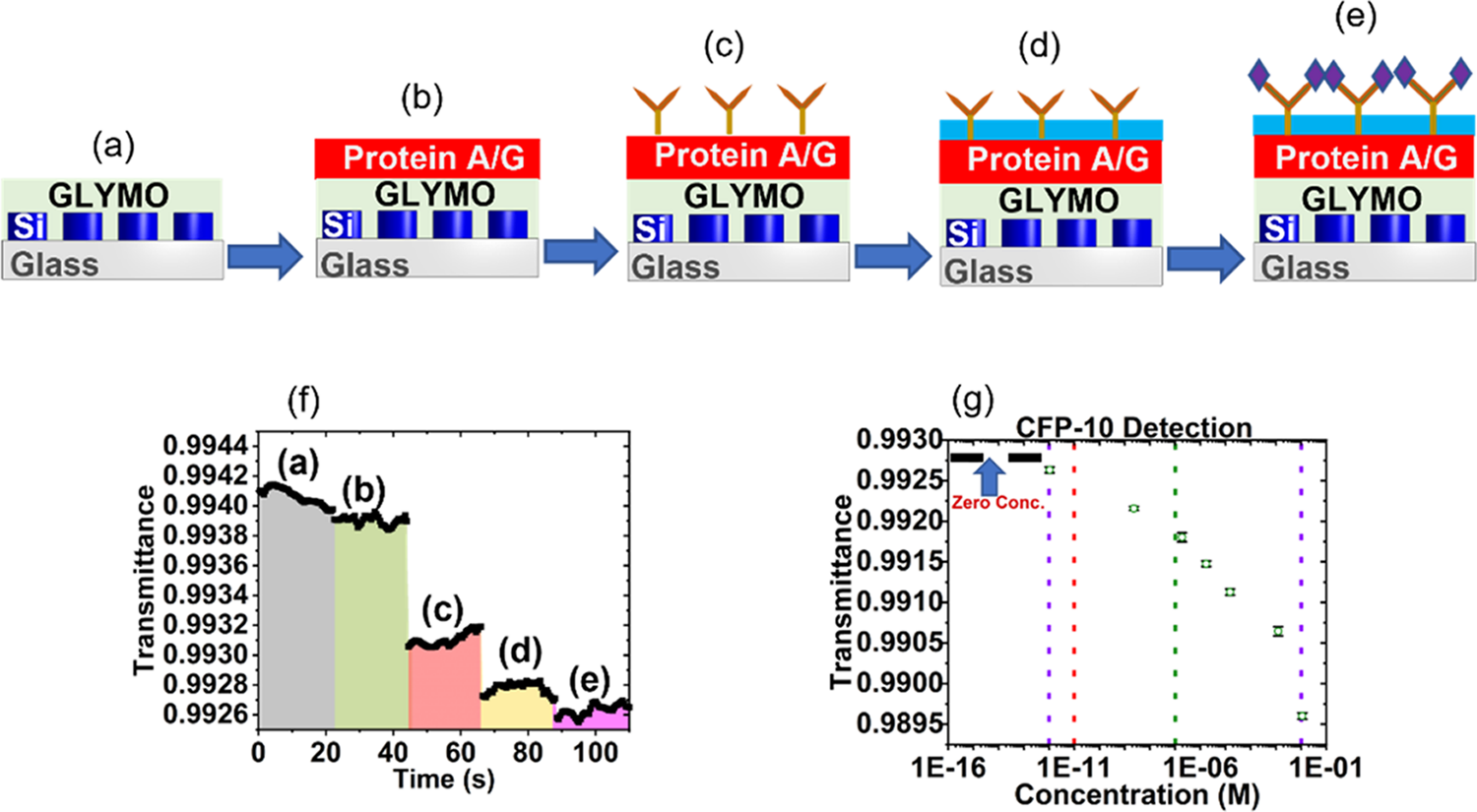
Functionalized metasurface platform: (a) silicon metasurfaces on a glass substrate with GLYMO added to bind proteins to Si and SiO_2_; (b) protein A/G is added, which enhances the binding affinity of human IgG (the capture antibody); (c) the “Y-shaped” structures are capture antibodies (anti-CFP-10 antibody) that serve as antigen-binding sites; (d) the blue layer represents a blocking buffer, which reduces the noise introduced by unspecific binding; and (e) the purple rhombuses represent the CFP-10 peptide in the sample that have bound to the measurement chip. (f) Sensor transmittance vs time highlighting the data collection time frame for each layer added to the metasurface chip according to panels (a–e), with a CFP-10 concentration of 1.06 pM (1.7 pgmL^–1^) measured in this figure. Temperature fluctuation effects during measurement are nullified by postmeasurement averaging. (g) Sensor transmittance versus CFP-10 peptide concentration after averaging. Here, we identify the dynamic range as 1 pm to 10 mM (purple dashed lines), the LOD (IC_10_) as 10 pM (red dashed line), and the sensitivity (IC_50_) as 0.1 μM (blue dashed line). Zero antigen concentration is represented by the black horizontal dashed line in the low-concentration region of the plot.

We use the bioassay described in the Experimental Section to measure a wide range of concentrations of the TB antigen CFP-10 (one of the top two biomarkers for detecting TB)^47^ in a phosphate buffer solution (PBS). The metasurface-based sensor produces the results seen in Figure 7f,g. Specifically, we are interested in identifying the dynamic range, the LOD, and the sensitivity of this CFP-10 peptide measurement. The dynamic range is the measured region of concentrations where we can identify distinct changes in transmittance. In our current data set, we measure a dynamic range of 11 orders of magnitude, spanning from 1 pM (1.6 pgmL^–1^) to 10 mM (16.0 mgmL^–1^). We use the standard IC_10_ metric that sets the LOD as a 10% saturation of the dynamic range.^13^ This places our LOD at 10 pM, which corresponds to 16.0 pgmL^–1^. This indicates that the obtained LOD value is several orders of magnitude more sensitive compared to standard ELISA measurements.^48^ Similarly, we denote the sensitivity as the IC_50_ value or a 50% saturation of the dynamic range.^13^ On our standard curve, this equates to 0.1 μM (160.3 ngmL^–1^). This proof-of-concept demonstration is for CFP-10 suspended in a homogeneous PBS solution, and performance will certainly change with samples spiked in commercialized human serum and clinical samples.^47^ However, with future improvements in sample filtration,^49,50^ optimization of antibody–antigen pairing,^51^ and recycling steps, this sensor is expected to show similar functionality for more complex human samples.

## 5 Conclusions

We have developed and characterized a compact and portable metasurface-based refractive index sensor applicable for bulk and surface detection. The final prototype cost represents an 87–96% cost reduction over existing detection methods offering comparable sensitivity. The measured LOD for bulk fluid refractive index measurements of 1.9 × 10^–6^ RIU rivals state-of-the-art dielectric-based photonic refractive index measurement devices. Metasurface full-wave model results have been replicated experimentally to verify nanoantenna array functionality. Biomolecule surface detection is demonstrated for the CFP-10 peptide, a tuberculosis biomarker. We measure an IC_50_ maximum sensitivity of 0.1 μM and an IC_10_ detection limit of 10 pM. The entire sensor described here may be further integrated into a photonic chip, making it deployable in smartphones and handheld devices to aid with preventative diagnostics and clinical monitoring. Although this platform was designed and fabricated to operate in the near-infrared regime, similar Huygens nanodisk arrays can be employed for portable sensors in the visible regime using a different material platform.^52^ By incorporating a more precise data logger and signal averaging, the limit of detection for this sensor could be further improved by 2 orders of magnitude at an additional cost of less than 50% of the current overall assembly cost. Ultimately, this platform shows promise for further improvements in sensitivity, cost, and size reduction, for applications in industrial process monitoring, infectious disease diagnostics, trace gas detection, and more.

## Supporting Information Available

Table S-1 shows geometry values of the metasurfaces designed and fabricated on the sensor chip; Figure S-1 illustrates the spectral behavior of Mie and asymmetric resonance metasurfaces as a function of the encapsulant refractive index; details about the sensor chip micro- and nanofabrication processes including images of the fabricated nanoantennas and the microfluidic chips, as shown in Figure S-2; Figure S-3 shows the schematic and description of the portable sensor circuitry; the bioassay development and test protocol details for detecting the CFP-10 peptide is included; and Table S-2 details the sensor bill of materials with the quantity and cost of parts purchased and assembled for the portable sensor (PDF)
