# Supplementary material for: Silicon Nanodisk Huygens Metasurfaces for Portable and Low-Cost Refractive Index and Biomarker Sensing
Source: ACS Appl Nano Mater. 2022 Mar 16;5(3):3983–91. doi: 10.1021/acsanm.1c04443 (PMC8961735; doi:10.1021/acsanm.1c04443)
Supplement: Supplementary file 1 — an1c04443_si_001.pdf [file an1c04443_si_001.pdf]

Supporting Information

Silicon Nanodisk Huygens Metasurfaces for Portable and Low-Cost Refractive Index and Biomarker Sensing

Isaac O. Oguntoye[1,§,*], *Brittany K. Simone*[1,§], *Siddharth Padmanabha*[1], *George Z. Hartfield*[1], *Pouya Amrollahi*[2], *Tony Y. Hu*[2], *Adam J. Ollanik*[1,3], *Matthew D. Escarra*[1]

[1]Department of Physics and Engineering Physics, Tulane University, New Orleans, Louisiana 70118

[2]Center of Cellular and Molecular Diagnosis, Tulane University, New Orleans, Louisiana, 70112

[3]Department of Physics, University of Colorado Boulder, Boulder, CO 80309

[*]Email: ioguntoye@tulane.edu

[§]I.O. and B.S. contributed equally to this paper

This supporting information document includes details about the 1.) geometry of the metasurfaces designed and fabricated on the sensor chip; 2.) spectral behavior of asymmetric resonance metasurfaces as a function of encapsulant refractive index; 3.) details about the sensor chip micro and nanofabrication processes; 4.) schematic and description of the portable sensor circuitry; 5.) bioassay development and test protocol details for detecting CFP-10 peptide; 6.) sensor bill of materials with the quantity and cost of parts purchased and assembled for the portable sensor.

Section 1: Metasurface Dimensions

Dimensions	Mie Resonance Nanodisks	Asymmetric Resonance Nanocylinders
Height	190 nm	190 nm
Diameter	330 nm	240 nm
Edge to Edge Spacing	251 nm	415 nm

Table S-1**:** Modeled dimensions of metasurfaces

Section 2: Metasurface resonance sensitivity to encapsulant index variations

The Huygens metasurfaces used in this work are distinguished by the type of resonances they excite. In the Mie resonance metasurfaces, the electric dipole resonance is more sensitive to changes in the encapsulant fluid refractive index compared to the magnetic dipole resonance. In the asymmetric resonance metasurfaces, the magnetic field and electric field resonances shift at nearly equal rates. These dual resonance designs lead to enhanced sensitivity of the metasurfaces due to the contribution of multiple resonances.

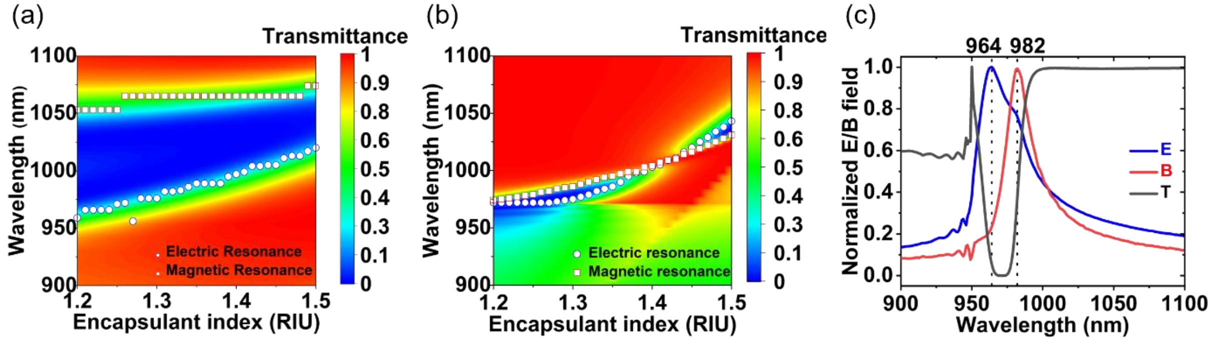

Figure S-1 (a). Metasurface transmittance (color map) as a function of incident wavelength and encapsulant refractive index showing that the electric field resonance (blue circles) is more sensitive to changes in the encapsulant index than the magnetic field resonance (red squares) (b).

Resonance shifts from varying encapsulant fluid refractive index for asymmetric resonance metasurfaces. (c). Electric and magnetic field amplitude vs. incident light wavelength at a fixed encapsulant fluid index (R.I. = 1.333) showing electric field and magnetic field asymmetric resonances at 964 nm and 982 nm respectively.

Section 3: Metasurface Micro and Nanofabrication

The amorphous silicon was grown on glass using electron beam evaporation and patterned into metasurfaces using the RAITH Voyager 100 electron beam lithography tool. This was followed by reactive ion etching using SF_6 and C_4F_8 gases at 10 mTorr and at flow rates of 33 sccm and 57 sccm using ICP (1000 W) and RIE (110 W) power sources respectively for feature definition. The metal mask is fabricated using photolithography and electron beam deposition of chromium. Microfluidic channels are patterned using photolithography onto a silicon wafer and cast into polydimethylsiloxane (PDMS). This segment is then bonded to the metasurface chip using a two-step process involving 1) surface activation via oxygen plasma bonding and 2) microfluidic channel positioning. For microfluidic channel positioning, deionized water is added onto the glass substrate and the microfluidic channel is placed and adjusted accordingly. The chip is then placed on a hotplate at 60°C for 20 minutes to enhance evaporation of the water droplets and fix the microfluidic channels into position.[1]

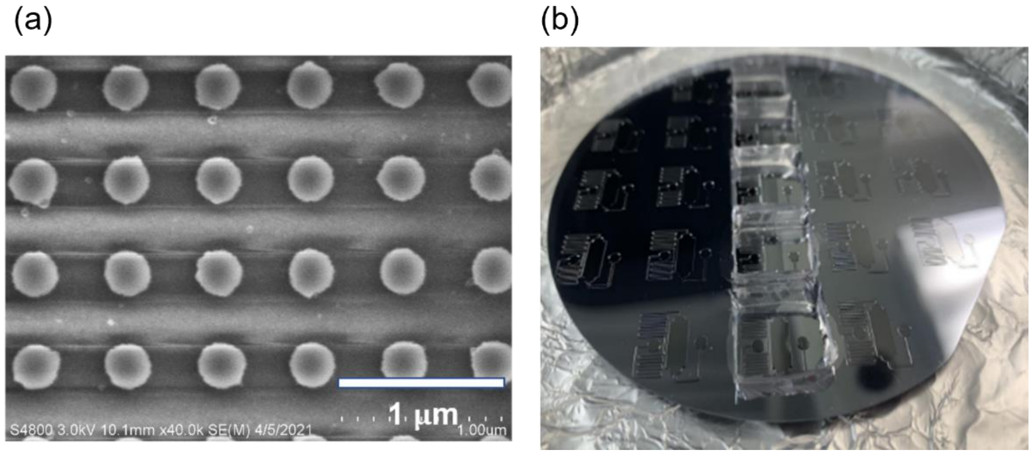

Figure S-2: (a). Scanning electron micrograph of fabricated sensor chip. (b). Image of fabricated microfluidic channels embedded in PDMS on a 4-inch silicon wafer.

Section 4: Circuit Design and Methodology

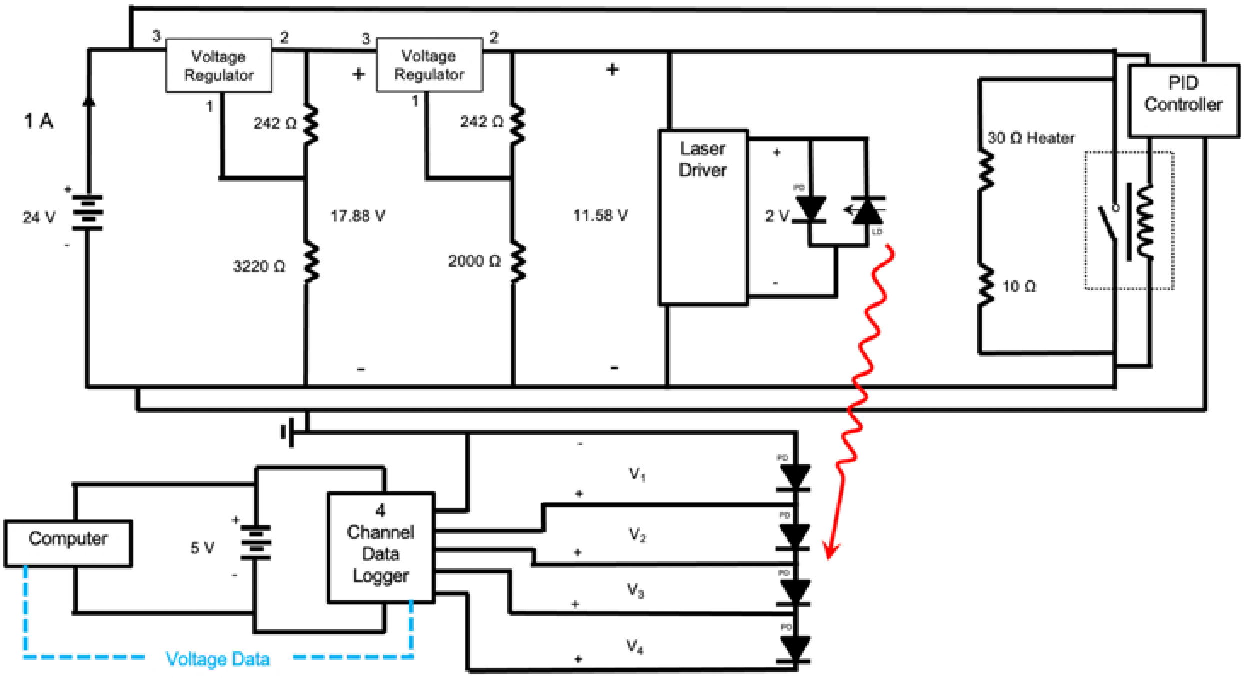

Figure S-3: Schematic showing circuitry for sensor operation. A stack of voltage regulators and dividers as well as the laser driver is used to fix the output power of the laser beam. The laser beam is incident on the measurement chip and the transmitted signal is collected on a 4-quadrant

photodetector represented by V_1 – V_4. These are collected by the 4-channel datalogger connected to a computer for on demand viewing and data analysis.

Section 5: Bioassay Development and Test Protocol

Preparation of the surface-functionalized testing platform deviates from the aforementioned protocol before oxygen plasma bonding to the PDMS channels. Here, we clean the surface with acetone and IPA then use polyimide conductive tape to block off everything outside of the chromium reflectance mask to prevent binding. We then spin coat a 4% [3-(2,3-Epoxypropoxy)propyl]trimethoxy silane (GLYMO)-96% deionized water solution onto the surface to create a uniform layer over the metasurfaces and reference channel. The chip then incubates on a 90ºC hot plate for two hours. This allows the water to evaporate and the GLYMO to bind to the surface of the sensor (Si and SiO_2). This chip is then washed with deionized water to remove any unbound chemical and the conductive tape is removed to expose the glass surface for oxygen plasma bonding. Plasma bonding is completed in the same manner as before, and then the chip is introduced to the housing and aligned using the same protocol in the sensor platform design section of the manuscript. After this is complete, the first optical measurement is made using a Phosphate Buffer Solution (PBS) fluid as the bulk. Protein A/G in a NaOH-DI solution with a pH ~9 is introduced to the channel and left to incubate at room temperature for 24 hours. After incubation, the chip is washed with PBS and a second measurement is taken. Anti-CFP10 Immunoglobin G (IgG) is introduced and serves as the capture antibody for this assay. IgG incubates for 1 hour at 37ºC before washing and measuring in PBS. The final stage of the assay is a blocking buffer agent that coats the unbound protein A sites and reduces the ability for undesired biomolecular compounds from binding to the surface and adding noise to the measurement. In this case, we are incubating the chip with SuperBlock™ for 2 hours at 37ºC. After washing the surface

with PBS, a final measurement is made in PBS, to serve as our 'zero concentration' baseline for the next meaurements. At this point, the sample (CFP-10 peptide, a marker for human tuberculosis) is introduced at a very low concentration. The sample is allowed to incubate for 1 hour at 37ºC and then washed with 0.05% Tween in PBS (PBST) and measured in PBS solution. PBST acts as a detergent that removes all unbound molecules. The sample measurement stage is repeated while concentration is increased to build up a standard curve of concentration versus transmittance.

Section 6: Sensor Bill of Materials

Component	Purpose	Vendor	Part #	Amount	Unit Cost ($)	Total Cost QTY1 ($)
**Quadrant Photodiode**	Detector	Mouser	QP5.8-6-TO5	1	44.54	44.54
**Transistor Socket**	Interface with detector	DigiKey	ED2152-ND	1	1.84	1.84
**Laser Diode**	Laser	Thorlabs	L980P010	1	28.95	28.95
**Adjustable Collimation Tube**	Collimate laser	Thorlabs	LTN330-B	1	244.79	244.79
**ESD Protection & Strain Relief Cable**	Electrical protection for laser	Thorlabs	SR9A-DB9	1	55.95	55.95
**Temperature PID Controller**	Controls temperature	Omega	CN16D3-M-DC	1	85.00	85.00
**USB Data Logger**	Records voltage	CAS Dataloggers	-	1	2,665.00	2,665.00
**Custom Enclosure**	Seal out light	Protocase	-	1	218.05	218.05
**Corner Machine Bracket**	Sample door	McMaster	2313N15	1	9.80	9.80
**Steel Corner Bracket**	Mount drawers	McMaster	1556A24	2	0.43	0.86

**Flat-Surface Machine Bracket**	Mount drawers	McMaster	2312N33	1	5.11	5.11
**Miniature Drawer Slides**	Access sample	McMaster	3351N11	1	26.13	26.13
**Tubing Union Assembly**	Access tubing	Idex-hs	P-630	2	10.99	21.98
**Quick-Turn Tube Coupling**	Syringe connection	McMaster	51525K317	1	15.48	1.55
**Vibration-Damping Mount**	Feet for enclosure	Mcmaster	60525K21	4	4.13	16.52
**Dovetail Rail 6"**	Align optics	Thorlabs	RLA0600	1	45.72	45.72
**Dovetail Rail Carrier**	Mount optics	Thorlabs	RC1	2	26.94	53.88
**1/2" Post Holder 1" Length**	Mount optics	Thorlabs	PH1	2	7.24	14.48
**Optical Post 1" Length**	Mount optics	Thorlabs	TR1	2	4.88	9.76
**Cage Plate 1" Dia.**	Mount laser	Thorlabs	CP33	1	16.39	16.39
**Mirror Mount**	Mount detector	Thorlabs	MFM10	1	26.52	26.52
**Laser Mount Adapter**	Mount laser	Thorlabs	AD15F	1	33.00	33.00
**SCR**	Relay for PID	Mouser	TN3015H-6T	1	1.20	1.20
**Circuit Board**	Connect electronics	McMaster	1305N11	1	6.96	6.96
**PCB Standoffs**	Mount PCB	Mcmaster	91443A220	1	5.05	2.02
**USB Adapter**	Access data logger	McMaster	1423N8	1	23.04	23.04
**USB Cord**	Data logger-computer	McMaster	4974T77	1	16.67	16.67
**Barrel-Style DC Connector**	Access power supply	Mcmaster	8320N118	1	1.06	1.06

**Adapter Cord**	AC to DC	McMaster	70235K934	1	14.84	14.84
**Friction Hinge**	For door	McMaster	1467A4	1	3.60	3.60
**Constant Power LD Driver**	Power laser	Thorlabs	LD1100	1	98.75	98.75
**U-Channel, Aluminum 6063**	Mount heater & stage	McMaster	9001K802	1	8.99	8.99
**Z-Bar, Aluminum 6061**	Hold sample	McMaster	7062T11	1	3.16	0.79
**Miniature Snap-Acting Switch**	Interlock	McMaster	7779K12	1	4.93	0.00
**Voltage Regulator**	Power heater & laser	Amazon	L7812CV	1	6.49	2.60
**Black out foam**	Light-tight edges	Amazon	R534H	1	2.93	0.293
**Black Acrylic Paint**	Anti reflection	Walmart	21885EX	1	0.50	0.01
**Relay**	Heater Circuit	Mouser	OSA-SH-224DM5	1	2.41	2.41
**Tygon Tubing**	Microfluidics	Darwin-Microfluidics	LVF-KTU-13	1	73.79	2.24
**24-gauge wire**	Electronics	DigiKey	2183-2634-ND	1	4.75	0.16
**Electrical Headers**	Electonics	DigiKey	528-2947-ND	4	1.25	5.00
**Black Foam Core Board**	Light Tight	Uline	n/a	1	4.40	0.09
**1/4"-20 Screws**	Mounting Optics	Thorlabs	SH25S038	1	8.27	0.66
**8-32 Screws**	Sample Stage	Thorlabs	SH8S025	1	6.65	0.27
**4-40 Screws**	Sample Stage	Thorlabs	SH4S038	1	8.87	0.71
**Washers**	Sample Stage	Thorlabs	W8S038	1	3.59	0.25

**4-40 Nuts**	Sample Stage	Grainger Industries	2GA51	1	2.09	0.04
**12.7 mm Dovetail Translation**	Stage motion control	Thorlabs	DT12/M	2	84.14	168.28
**Wafer-scale nanoimprint Lithography**[2]	Sensor Platform			1	7.50	7.50
**TOTAL PER UNIT**						3,994.25

References

1. Kim, J. Y.; Baek, J. Y.; Lee, K. A.; & Lee, S. H. Automatic aligning and bonding system of PDMS layer for the fabrication of 3D microfluidic channels. *Sensors Actuators A* **2005** 119 593–598. doi:10.1016/j.sna.2004.09.023.
2. Sreenivasan, S. V.; Willson, C. G.; Schumaker, N. E.; & Resnick, D. J. Low-cost nanostructure patterning using step and flash imprint lithography. *Nanostructure Sci. Metrol. Technol.* **2002** 4608 187–194. doi:10.1117/12.437804.
